# To what extent does hyaluronic acid affect healing of xenografts? A histomorphometric study in a rabbit model

**DOI:** 10.1590/1678-7757-2017-0004

**Published:** 2018-01-16

**Authors:** Osman Fatih Arpağ, Ibrahim Damlar, Ahmet Altan, Ufuk Tatli, Ahmet Günay

**Affiliations:** 1 Mustafa Kemal University, Faculty of Dentistry, Department of Periodontology, Hatay, Turkey; 2 Private Practice, Department of Oral and Maxillofacial Surgery, Hatay, Turkey; 3 Gaziosmanpasa University, Faculty of Dentistry, Department of Oral and Maxillofacial Surgery, Tokat, Turkey; 4 Cukurova University, Faculty of Dentistry, Department of Oral and Maxillofacial Surgery, Adana, Turkey; 5 Dicle University, Faculty of Dentistry, Department of Periodontology, Diyarbakır, Turkey

**Keywords:** Hyaluronic acid, Xenograft, Histological technique, Bone formation

## Abstract

Among the many graft materials that have been used for the treatment of bone defects in oral and maxillofacial regions is xenograft. To improve osteoconductive effects of xenografts, they have been combined with various biocompatible materials, such as hyaluronic acid and bone morphogenetic protein.

**Objective:**

To determine bone-healing capacity of high molecular weight hyaluronic acid (HA) combined with xenograft in rabbit calvarial bone defects.

**Material and methods:**

Ten adult male New Zealand rabbits (mean weight 3 kg) were included in the study. Three 6-mm-diameter bicortical cranial defects were created on calvarial bone of all rabbits. These defects were filled as follows: a) xenograft; b) HA+xenograft; c) autograft. One month after the first operation, rabbits were sacrificed. Specimens were evaluated histomorphometrically.

**Results:**

Considering multiple comparisons, differences regarding new bone were statistically significant between all groups (p<0.05). The volume of residual graft was significantly decreased in HA group compared to xenograft group (p=0.035). Marrow space, trabecular thickness (TbTh), trabecular width (TbWi), trabecular separation (TbSp), and number of node: number of terminus (NNd:NTm) in the autograft group were significantly better than xenograft and HA groups (p<0.05). However, regarding marrow space, TbTh, TbWi, TbSp, and NNd:NTm values, xenograft and HA groups showed similar results and the difference were not significant (p>0.05).

**Conclusion:**

These results support that high molecular weight hyaluronic acid could contribute to the healing of xenograft by improving the percentage of new bone formation and reducing the percentage of residual graft. However, HA did not significantly affect the quality of newly formed bone assessed by microarchitectural parameters.

## Introduction

An understanding of the mechanisms of bone repair and regeneration is basic to bone defect treatment, alveolar socket healing and dental implant surgery^2^. Thus, various graft materials such as autografts, allografts, xenografts, and alloplasts have been used in clinical practice^32^. Although autogenous bone grafts are considered the gold standard, they have many disadvantages, including donor site morbidity, inadequate amount of graft, and extensive surgery time^20^.

Xenografts are proper alternatives for bone repair and regeneration because of their similarity to human bone^28^. Although the available amount of autograft material is always limited, one can obtain as much xenograft as desired. Due to hydroxylapatite structure, natural bovine bone is potentially a much better graft material than a synthetic bone substitute^1^. However, unresorbed graft remnants of bovine bone have been observed in histological analyses even after three years^14^. Because xenografts are osteoconductive rather than osteoinductive, it is important to identify methods to improve their effectiveness *in vivo* ^27^.

Hyaluronic acid (HA) is a high molecular weight polysaccharide that is also non-sulfated glycosaminoglycan that is distributed widely throughout connective tissue, synovial fluid and extracellular matrix of other tissues. HA plays an important role in various biological cycles, including wound healing, chondrogenesis, immune response and cell migration^4^ ^,^ ^5^. In addition, HA affects the early biochemical and physiological stages of osteogenesis as continuous extracellular matrix component in bone morphogenesis^23^. Sasaki and Watanabe^24^ (1995) demonstrated that HA could accelerate new bone formation by facilitating the differentiation of mesenchymal cells. In another study, HA combined with autogenous bone graft accelerated new bone formation in periodontal infrabony defects in nine patients^3^. In the literature, combinations of HA with different graft materials have been evaluated regarding bone-healing capacity *in vivo* and *in vitro* ^3^ ^,^ ^5^ ^,^ ^11^ ^,^ ^25^. However, we could not find any study showing the effects of HA on xenogenic graft healing in an animal model.

Our hypothesis is that high molecular weight HA combined with a xenograft could improve bone healing. Therefore, the aim of this study was to experimentally determine the bone-healing capacity of xenografts when combined with HA in rabbit calvarial bone defects, as measured by histomorphometric parameters.

## Material and methods

**Figure 1:**
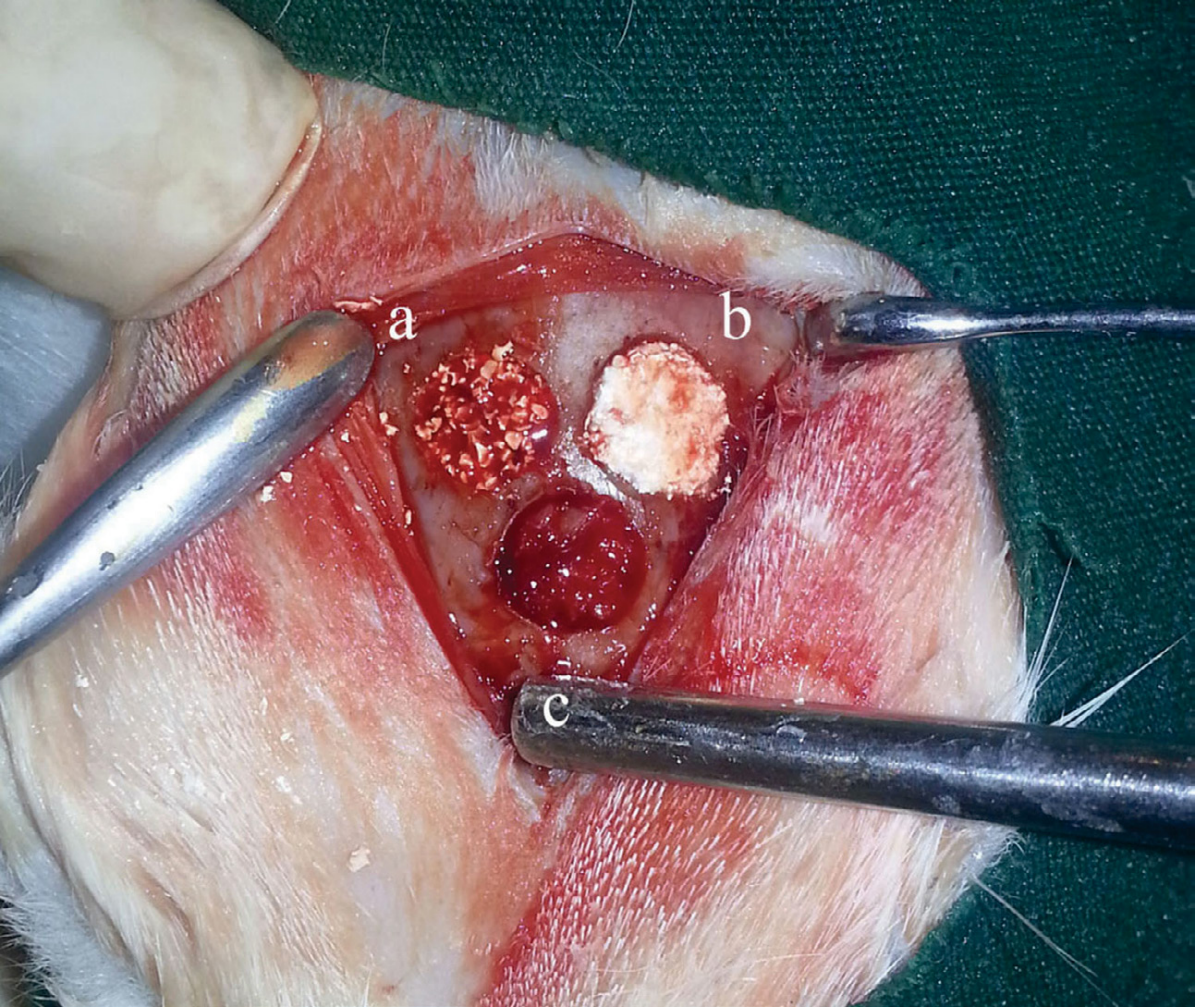
Experimental materials were grafted as follows: (a) xenograft; (b) xenograft + HA; (c) autogenous bone

This study was conducted in the Experimental Research Centre in Mustafa Kemal University. Animal ethics committee of Mustafa Kemal University approved all experiments before beginning the study (Ethical approval number: 2013-7/15). Ten adult male New Zealand rabbits (mean weight 3 kg) were included for this study. General anaesthesia was performed by using 35 mg/kg Ketamine (Ketalar®, Pfizer, Istanbul, Turkey) and 3 mg/kg Xylazine (Rompun®, Bayer, Istanbul, Turkey) intramuscularly. To provide haemostasis and local anaesthesia, 2 ml articain was supraperiostally injected into the surgical area, which was, subsequently, shaved and stained with iodine solution to avoid any bacterial contamination. We exposed the calvarial bone (occipital, frontal, and parietal bone) by making a full-thickness linear incision from nasal bone to mid-sagittal crest. Afterwards, we created three 6-mm-diameter bicortical cranial defects by using trephine bur under copious irrigation with sterile saline solution. To prevent damage to the durameter, first we created a portion of cavity depth with the trephine bur and afterwards we removed the remaining portion of the cavity with the diamond round bur. In conclusion, three bicortical defects were obtained. These defects were filled as follows:

1. Xenogenic graft (Integros BonePlus®, Integros, Adana, Turkey) (xenograft group);
2. Xenogenic graft+HA (Orthovisc® 2 ml, Biomeks, Istanbul, Turkey) (xenograft+HA group);
3. Autogenous bone graft (beneath the other defects) (autograft group) (Figure 1).

The autogenous bone graft was obtained from the calvaria portion previously removed to create the bone defect. Before the defect was filled, the autogenous bone graft had been crushed. After placement of the experimental materials, all defects were covered with a 22×22-mm resorbable collagen membrane (Collagene At®, Centro Di Odontoiatria Operativa SRL, Padova, Italy), and the surgical area was sutured with 4/0 resorbable suture (Vicryl®, Johnson&Johnson, Brussels, Belgium). The same operator performed surgical procedures. To avoid postoperative infection and pain, an analgesic (diclofenac sodium) and an antibiotic (cefazolin 25 mg/kg) were intramuscularly injected twice a day for four days. Animals were housed in separate cages and underwent normal diet programme. Their body weight and food intake were monitored and recorded day newly formed bone tissue to day. One month after the first operation, rabbits were sedated by the same general anaesthesia method and sacrificed by an intracardiac injection with 100 mg/ kg sodium thiopental (Pental®, Bilim, Istanbul, Turkey) and, afterwards, the calvarial bones were excised. The calvarial bone blocks that belonged to the same groups were transferred in numbered boxes and recorded according to the study groups. The investigator who performed the histomorphometric analyses was strictly unaware of these records.

### Histomorphometric analysis

After the excision of calvarial bones, we separated the experimental sites using a steel saw. Samples were fixed in 10% formalin solution. Then, they were dehydrated in ethyl alcohol solution in concentrations ranging between 70% and 99% for 15 days and then embedded in acrylic resin blocks (Technovit® 7200, Heraeus Kulzer GmbH, Wehrheim, Germany). By an electric diamond saw and an emery, three serial 40-μm transverse sections were created and stained with toluidine blue *per* sample. After samples were photographed by a digital camera (Olympus® DP 70, Tokyo, Japan) and the microscope was adapted to at a magnification rate of 4x, we transferred the obtained views to the computer and performed measurements by using the histomorphometry software (WinTAS version 0.1, University of Leeds, UK). For each parameter, the mean values of three sections were assumed for the corresponding sample. Considering histomorphometric analysis, histologic (tissue compartments) and microarchitectural parameters were evaluated. The nomenclature and calculations for bone histomorphometry were applied in accordance with the report from the American Society for Bone and Mineral Research^9^.

### Histologic parameters (tissue compartments)

Histologic parameters were percentages of new bone, marrow space, and residual graft amount. The new bone parameter indicates the ratio of current bone to total tissue volume at the analyzed section. The residual graft parameter indicates the ratio of residual graft to total tissue volume at the analyzed section. The marrow space parameter indicates the ratio of soft tissue (rest of bone and residual graft amount) to total tissue volume at the analyzed section

### Microarchitectural parameters

Microarchitectural parameters were trabecular thickness (TbTh), trabecular width (TbWi), trabecular separation (TbSp), and node:terminus ratio (NNd:NTm) of new bone. TbSp is defined as the distance between the edges of the trabeculae. TbWi is defined as the mean width of the edges of the trabeculae. TbTh is defined as the mean thickness of the edges of the trabeculae, which we calculated by the histomorphometry software using a mathematical equation with a correction factor for section obliquity. While the TbTh is the distance provided from three-dimensional imaging of the bone, the TbWi is the distance obtained from the same region in two-dimensional images of the bone. Although thickness and width are identical in a numeric manner, TbTh is calculated by dividing TbWi by 4/p or 1.2^7^ ^,^ ^9^. The ratio between the numbers of nodes and termini in a section is an index of the spatial connectivity in the trabecular network. Except for NNd:NTm, which was expressed in ratio, all parameters were expressed in μm.

### Statistical analysis

Data were analysed using MedCalc statistical software (version 10.1.6, Mariakerke, Belgium). As data were not normally distributed, the Kruskal- Wallis one-way analysis of variance and the Mann- Whitney U test were utilised. When employing multiple comparisons, we corrected p-values using the Bonferroni adjustment procedure. In all statistical tests, significance level was defined as p<0.05.

## Results

**Figure 2:**
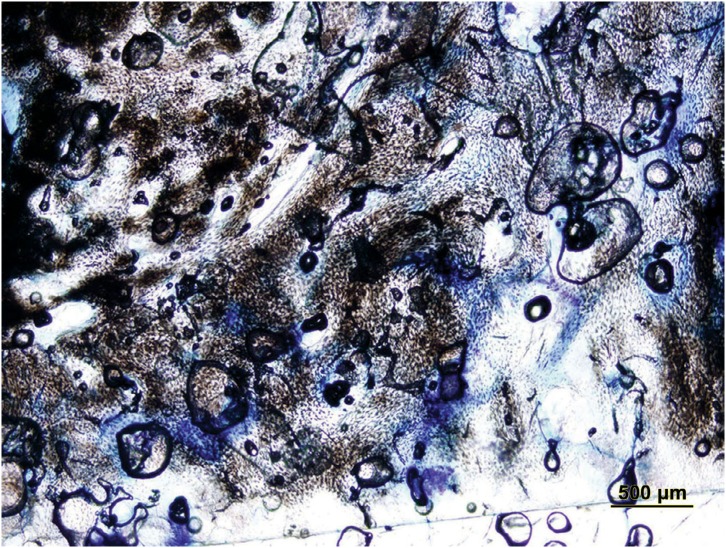
Histological transversal section stained with toludine blue in autograft group (original magnification 4×)

**Figure 3:**
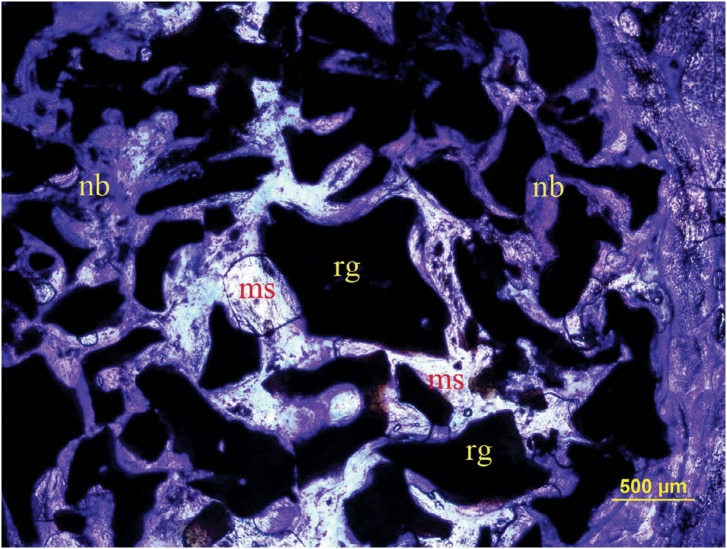
Histological transversal section stained with toludine blue in xenograft + HA group (original magnification 4×). nb: new bone; ms: marrow space; rg: residual graft particles

**Figure 4:**
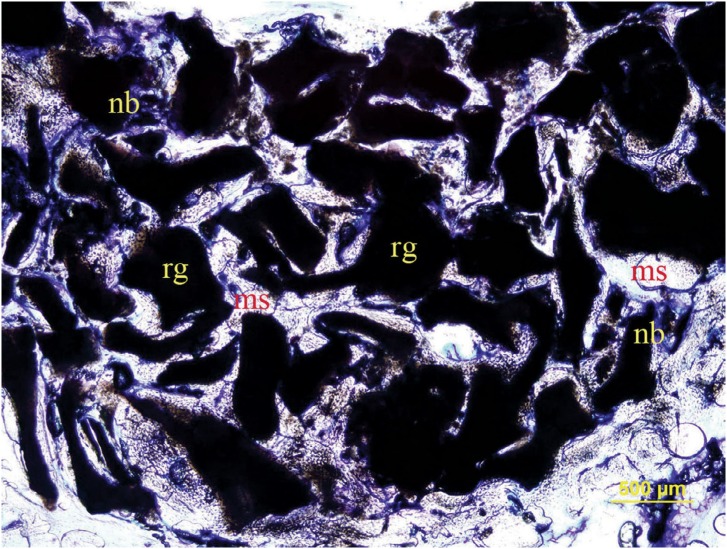
Histological transversal section stained with toludine blue in xenograft group (original magnification 4×). nb: new bone; ms: marrow space; rg: residual graft particles

After surgery, all rabbits recovered well and the expected increase in weight during the post-surgical period was recorded. Complications, such as paralysis, convulsions, respiratory distress, and wound infections were not observed. Results of histomorphometric comparisons among groups are shown in Table 1. Regarding percentages of tissue compartments and microarchitectural parameters, significant differences were observed among study groups (p<0.05). Considering multiple comparisons, differences regarding new bone between all groups were also statistically significant (p<0.05). New bone value was best in the autograft group (p<0.05). New bone value of the xenograft+HA group was also significantly greater than the xenograft group (p<0.05). The volume of residual graft was significantly decreased in HA group compared to xenograft group (p=0.0355) (Figures 2, 3, and 4).

**Table 1:** Histomorphometric comparisons of the study groups. Concerning “tissue compartments”; percentages of new bone, marrow space, and residual graft materials are shown. Concerning “microarchitectural parameters”; trabecular thickness, trabecular width, trabecular separation, and node:terminus ratio values of the newly formed bone are shown. Results are presented as means ± standard deviations (SD)

	Groups (Mean±SD)			p
	Autograft (n=10)	Xenograft (n=10)	Xenograft+HA (n=10)	
**Tissue compartments (%)**				
New bone	75.31±2.88ᵃ	26.92±4.49^c^	30.72±3.14^b^	0.0001
Marrow space	24.67±2.88ᵃ	11.01±4.63^b^	14.49±4.41^b^	0.0001
Residual graft	0	63.59±7.18^b^	54.78±5.16ᵃ	0.0355
**Microarchitectural parameters (μm)**				
Trabecular thickness (TbTh)	38.19±5.26ᵃ	16.93±2.11^b^	20.12±7.28^b^	0.0001
Trabecular width (TbWi)	42.35±6.43ᵃ	18.82±2.48^b^	24.12±8.73^b^	0.0001
Trabecular separation (TbSp)	2.71±0.69ᵃ	4.96±1.55^b^	4.51±1.35^b^	0.0006
Node:Terminus ratio (NNd:NTm)	6.99±2.57ᵃ	3.49±1.36^b^	3.86±0.78^b^	0.0020

Different letters in a row show statistically significant differences between three groups. Order of importance a>b>c (if any)

Marrow space, TbTh, TbWi, TbSp, and NNd:NTm in the autograft group were significantly better than in xenograft and xenograft+HA groups (p<0.05). However, regarding marrow space and microarchitectural parameters including TbTh, TbWi, TbSp, and NNd:NTm values, xenograft and xenograft+HA groups showed similar results and no significant difference (p>0.05).

## Discussion

In this study, we aimed to examine the effects of high molecular weight HA on xenograft bone material regarding new bone formation and the bone microarchitecture in rabbit calvarial defect model.

Anatomical and physiological properties of rabbit calvaria are similar to human bone. Owing to poor blood supply and absence of bone marrow, the calvaria is a proper testing area for the evaluation of bone repair and effectiveness of regenerative materials^16^ ^,^ ^19^. Material stability and host reactions can be examined to evaluate healing during a period of 2-4 weeks postinjury, whereas bone regeneration parameters such as bone remodelling, material resorption or amount of bone regeneration can be detected after 8 weeks or more of healing^26^. Jiang, et al.^15^ (2012) reported that a 5-mm defect size in rabbit calvaria model could not heal spontaneously over a period as long as 8 weeks. Therefore, we created the three 6-mm-diameter bicortical defects on the calvariae of the rabbits to test whether HA accelerates the healing of xenogenic graft material. These defects were filled with the following materials: xenograft; a combination of xenograft and high molecular weight HA; and autogenous graft. Our rationale for using high molecular weight HA in this study is that Sasaki and Watanabe^24^ (1995) reported that high molecular weight HA increased new bone formation in rat femoral defects after bone marrow ablation.

The treatment of craniomaxillofacial defects is one of the greatest challenges in bone healing. Autografts induce several bone-healing mechanisms, such as osteogenesis, osteoinduction, and osteoconduction^6^ ^,^ ^8^. Although xenografts are widely used in dentistry, they have a limited capacity for osteogenesis because of their low viable cell capacity. In addition, the chemical processes required to inhibit the transmission of animal diseases prevent xenografts from having osteoinductive effects^13^ ^,^ ^21^. Xenografts that have no osteogenic or osteoinductive effects lead to poor clinical results. However, modern tissue engineering technology makes it is possible to design new scaffolds and tissue grafts to improve osteogenic, osteoinductive, and osteoconductive effects and to mitigate certain disadvantages of these grafts^21^.

Owing to its regulatory role in bone and fracture healing, HA is used to increase scaffold production in tissue engineering^21^. In our study, the application of xenograft mixed with HA into the defect was easier than the xenograft alone; the HA gel provided a stable putty form, which kept graft particles together. The aforementioned advantages of this form have been confirmed by another study, in which putty form of various graft materials was used^25^.

It is crucial to fill craniomaxillofacial defects as soon as possible. Therefore, in this study, we investigated the effects of HA on new bone formation at one month. The rate of new bone formation in the autograft group was significantly higher than in other groups. Similarly, the combination of xenograft+HA resulted in statistically significantly greater bone fill than the xenograft alone. These results support our hypothesis that HA promotes new bone formation, most probably by increasing the osteoinductive effect of the xenograft. In this experimental study performed on rabbit calvaria, we can attribute the osteoinductivity of HA to induced growth factors, such as bone morphogenetic proteins^31^. Bone morphogenetic proteins are known to play important roles in the migration of progenitor cells, proliferation, and differentiation of mesenchymal cells to osteogenic cells, vascular invasion, and bone remodelling^18^.

To evaluate the healing capacity of 6-mm defects filled with anorganic bovine bone in rabbit calvaria, one month was sufficient regarding bone formation. Our hypothesis was compatible with the study of Paknejad, et al.^22^ (2014), which was performed with methods similar to those used in our study. They concluded that new bone formation occurred at four weeks when anorganic bovine bone was used alone. Several studies have used a mixture of HA and various bone graft materials, and some of these combinations increased new bone formation rates^12^ ^,^ ^24^.

We observed a significant decrease in the percentage of residual xenograft particles in the xenograft+HA group. This could be because the amount of xenograft particles *per* unit volume in the xenograft+HA group was less than that of the xenograft group. However, Zanchetta, et al.^33^ (2012) reported that the use of injectable HA gel in the marrow cavity of the femur could induce bone formation after one week. This effect of HA possibly provided new bone formation by resorbing xenograft bone substitute, which also has an osteoconductive effect within one month. In addition, when combined with xenograft, HA significantly increased space among graft particles, helping to decrease the packing density and allowing vascular and cellular entrance into the grafted area. Scaffolding materials such as HA are also known to have osteogenic and angiogenic potential because of their high vascular and cellular activity^11^.

In addition to new bone formation, which is an important indicator in the complete regeneration of bone defects, the microstructure and microarchitecture of newly formed bone are significant indicators of bone quality^10^ ^,^ ^17^. In this animal study, three different treatment modalities were compared, and obtained specimens were analysed regarding marrow space, trabecular thickness, trabecular width, trabecular separation, and node:terminus ratio, which are essential histomorphometric parameters of bone quality. A node in the network of bone tissue is a connection point of three or more trabeculae; however, a terminus is described as a point at which a trabecula is not joined to any other one. It has been supposed that the NNd:NTm is a way of explaining the connectivity of the trabecular network^30^.

In this study, the structures of bone microarchitecture and the marrow space of the autograft group were significantly better than in the other two groups. Differences between xenograft+HA and xenograft groups were not statistically significant. Even though groups exhibited similar results, we might conclude that HA had a slightly positive effect on the quality of the trabecular network in newly formed bone. The strength of the trabecular bone network is largely associated with the total bone mass, as well as bone microstructure and microarchitecture^29^.

## Conclusions

These results support our hypothesis that the high molecular weight HA can positively affect anorganic bovine bone healing concerning new bone formation in a short time; however, HA does not appear to significantly affect the structure of the trabeculae. There is a need for further studies to evaluate the microarchitecture of newly formed bone tissue during xenograft healing when combined with the high molecular weight HA.
